# Inspirations of Cobalt Oxide Nanoparticle Based Anticancer Therapeutics

**DOI:** 10.3390/pharmaceutics13101599

**Published:** 2021-10-02

**Authors:** Huanshao Huang, Jiajun Wang, Junai Zhang, Jiye Cai, Jiang Pi, Jun-Fa Xu

**Affiliations:** 1 Department of Clinical Immunology, Institute of Laboratory Medicine, Guangdong Provincial Key Laboratory of Medical Molecular Diagnostics, School of Medical Technology, Guangdong Medical University, Dongguan 523808, China; huanghuanshao@gdmu.edu.cn (H.H.); timmywjj@gdmu.edu.cn (J.W.); zhangjunai@gdmu.edu.cn (J.Z.); 2 Department of Chemistry, Jinan University, Guangzhou 510632, China; tjycai@jnu.edu.cn

**Keywords:** cobalt oxide nanoparticles, anticancer therapeutics, synthesis, anticancer functions, mechanisms

## Abstract

Cobalt is essential to the metabolism of all animals due to its key role in cobalamin, also known as vitamin B12, the primary biological reservoir of cobalt as an ultra-trace element. Current cancer treatment strategies, including chemotherapy and radiotherapy, have been seriously restricted by their side effects and low efficiency for a long time, which urges us to develop new technologies for more effective and much safer anticancer therapies. Novel nanotechnologies, based on different kinds of functional nanomaterials, have been proved to act as effective and promising strategies for anticancer treatment. Based on the important biological roles of cobalt, cobalt oxide nanoparticles (NPs) have been widely developed for their attractive biomedical applications, especially their potential for anticancer treatments due to their selective inhibition of cancer cells. Thus, more and more attention has been attracted to the preparation, characterization and anticancer investigation of cobalt oxide nanoparticles in recent years, which is expected to introduce novel anticancer treatment strategies. In this review, we summarize the synthesis methods of cobalt oxide nanoparticles to discuss the advantages and restrictions for their preparation. Moreover, we emphatically discuss the anticancer functions of cobalt oxide nanoparticles as well as their underlying mechanisms to promote the development of cobalt oxide nanoparticles for anticancer treatments, which might finally benefit the current anticancer therapeutics based on functional cobalt oxide nanoparticles.

## 1. Introduction

In the past few decades, cancer, a disease that is induced by the evasion of one’s own protective system against malignant cells, is causing continuing high morbidity and mortality worldwide. As is commonly known, radiotherapy, chemotherapy and immunotherapy are commonly used to fight cancer. However, like a double-edged sword, these methods can also produce lots of side effects while relieving cancer progress. For instance, the systemic cytotoxicity and severe side effects of radiotherapy are the biggest obstacle to their maximum therapeutic efficacy for cancer [1,2]. In addition, chemotherapy drugs, which always show poor selectivity, can also easily kill a large number of normal cells, destroy the immune system and develop drug resistance [3]. Although immunotherapy is attracting increasing attention worldwide, it is still extremely expensive and may also be life-threatening if the anticancer immune responses are not well controlled [4,5]. Based on the current status of cancer therapy strategies, there is an urgent need to develop new technologies to combat cancer.

As one of the trace elements, cobalt is closely associated with human health [6]. Cobalt is essential to the metabolism of all animals due to its key role in cobalamin, also known as vitamin B12, the primary biological reservoir of cobalt as an ultra-trace element. Besides vitamin B12, there are also some cobalt proteins, such as methionine aminopeptidase 2, an enzyme that occurs in humans and other mammals, and nitrile hydratase, an enzyme in bacteria that metabolizes nitriles, both of which play important roles in the metabolisms. Metal oxide nanoparticles have been a research hotspot for decades in different fields, including medicine. Based on the emerging biological roles of cobalt, cobalt oxide nanoparticles (including CoO NPs, Co_2_O_3_ NPs and Co_3_O_4_ NPs) have been applied in different fields because of their special physical and chemical properties [7,8,9]. Cobalt oxide has been widely used for electrochemical applications due to its abundance in nature, feasible synthesis, easy-to-customize morphology and good chemical properties [10,11]. Besides the production of batteries [12], cobalt oxide nanoparticles could also be used to produce capacitors and different kinds of biosensors [13,14,15,16,17]. Similarly, cobalt oxide nanoparticles might also play important roles in human health, thus showing potential for a variety of biomedical applications. In some infectious diseases, cobalt oxide nanoparticles exert powerful anti-infective capabilities, indicating their potential to act as antibacterial, antifungal and anti-parasite agents [18,19].

In recent years, the anti-tumor activity of cobalt oxide nanoparticles has also attracted more and more attention due to their selectivity against cancer cells. Our recent work also demonstrated that cobalt oxide nanoparticles showed high toxicity against cancer cells while the same concentrations would not induce cytotoxicity against normal cells [20]. Moreover, cobalt oxide nanoparticles were also found to exert anticancer effects by inducing apoptosis or causing DNA damage [21,22]. Last but not least, cobalt oxide nanoparticles could also act as delivery systems with drugs or antigens encapsulated for targeted cancer therapy, causing more selective cancer cell death or stimulating selective anticancer immune responses [23,24,25].

In this review, we aimed to summarize the synthesis of cobalt oxide nanoparticles and the research progress of cobalt oxide nanoparticles for cancer therapy, which we believe would extend the future research of cobalt oxide nanoparticles for anticancer treatment and benefit the development of novel anticancer strategies.

## 2. Synthesis of Cobalt Oxide Nanoparticles

Cobalt oxide nanoparticles show various activities based on their different sizes and shapes. Therefore, it is particularly important to control the size and shape of cobalt oxide nanoparticles during the preparation process according to different purposes.

### 2.1. Chemical Synthesis

Chemical precipitation is the most commonly used method for preparing cobalt oxide nanoparticles, which generally requires two kinds of reagents: cobalt precursor and precipitant solution. Generally, the cobalt precursor and the precipitant solution are mixed and stirred first, then centrifuged, dried to collect the precipitates and then heated to obtain cobalt oxide nanoparticles. Javed et al. synthesized cobalt oxide nanoparticles using cobalt chloride solution and oxalic acid solution [24]. For the preparation of sphere cobalt oxide nanoparticles, Raman et al. used a simple method to centrifuge a mixture of CoCl_2_·6H_2_O and sodium tartrate that was vigorously stirred for 6 h at room temperature to obtain a precipitate. Finally, the washed and dried powder was placed in a high-temperature muffle furnace at 580 to 600 °C in air for 2 h and then stored in the furnace until it was cooled to room temperature [26]. Moradpoor et al. used three levels of Co (NO_3_)_2_·6H_2_O and three levels of KOH stirred for different times to synthesize cobalt oxide nanoparticles with antibacterial activity. The obtained deposit was dried and calcined under specific conditions to obtain cobalt oxide nanoparticles [7]. In addition to the examples mentioned above, there are lots of researchers using chemical precipitation to synthesize cobalt oxide nanoparticles [10,11,19].

The biggest feature of cobalt oxide nanoparticle preparation by chemical synthesis is that it is simple and easy to implement. That is why lots of researchers choose this method to synthesize cobalt oxide nanoparticles. However, we cannot deny that the high temperature needed for cobalt oxide nanoparticle formation in chemical precipitation methods is a high-energy-consuming process. In addition, sometimes, it is difficult for some biological labs to finish the preparation processes due to the restricted reaction environments.

Cobalt oxide nanoparticles can also be produced by a simple chemical reduction method at room temperature, which is much more energy-saving than the chemical precipitation methods. Kanwal et al. added cobalt chloride and NaBH_4_ to the trisodium citrate solution, stirring constantly and maintaining the pH of the solution at 8 to collect cobalt oxide nanoparticles. Finally, the obtained powder was repeatedly washed with pure water and ethanol and dried in air at room temperature for 24 h [27]. This strategy provides a much easier method for cobalt oxide nanoparticle synthesis, which is not only energy-saving but also very easy to finish by conventional biological labs.

Thermal decomposition is also one of the chemical synthesis methods for cobalt oxide nanoparticle synthesis, which also requires high temperatures. Cobalt oxide nanoparticles can be synthesized by spinning the CoCl_2_·3H_2_O and Na_2_CO_3_ mixtures in a molar ratio of 1:1 for 1 h to obtain the nanoparticle precursor by centrifugation. Then, the precursor can be calcined in a ceramic crucible at 300 °C in air for 2 h to obtain cobalt oxide nanoparticles [23,25,28].

In addition, the template synthesis method, one of the most widely used methods for nanoparticle synthesis, can also be used for the synthesis of cobalt oxide nanoparticles. Kim et al. synthesized cobalt oxide nanoparticles using apo horse spleen ferritin (apoHoSF) with only a protein shell and no core as a template. In short, in an aqueous solution, the ferritin protein cage was used as a template to prepare hollow cobalt oxide nanoparticles by controlling the number of metal atoms in the ferritin [29]. The nanoparticles prepared by this method have good biocompatibility and are suitable for a variety of biomedical applications.

The immiscible solvents form emulsions under the action of surfactants, form nuclei in the “microbubbles”, coalesce, agglomerate and heat-treat to obtain nanoparticles. Dogra et al. prepared cobalt oxide nanoparticles using three metal surfactants, namely CoCTAC (Bishexadecyltrimethylammonium cobalt tetrachloride), CoDDA (Bisdodecylamine cobalt dichloride) and CoHEXA (bishexadecylamine cobalt dichloride). Cobalt oxide nanoparticles produced by this method show potential for antibacterial and anticancer treatments [30]. In general, the microemulsion method has the advantages of cheap raw materials, simple experimental equipment, easy operation, mild reaction conditions, controllable particle size and so on. It is widely used in the preparation of nanomaterials.

### 2.2. Chemical-Physical Synthesis

Dekkers et al. prepared cobalt oxide nanoparticles by the chemical-physical method, namely supercritical water hydrothermal synthesis. Firstly, the water pumped through the preheating coil (~400 °C) was brought into contact with the metal salt solution flowing at room temperature, while keeping the flow rate, temperature and pressure constant at 240 bar. Then the mixture that reached the mixing point was cooled immediately, and the pressure was reduced through the back pressure regulator to the ambient conditions. Finally, after centrifugation, washing and drying, cobalt oxide nanoparticles were obtained [31]. The synthesis of nanoparticles by supercritical hydrothermal synthesis technology has many special advantages. For example, the size of the nanoparticles is controllable and the distribution range is narrow. In addition, the preparation method is simple, and the energy consumption is low. More importantly, there is no organic solvent involved in the whole process, which is environmentally friendly. However, the product of the reaction requires appropriate separation techniques to extract.

### 2.3. Physical Synthesis

The principle of the physical synthesis of nanoparticles is mainly to use optical and electrical technology to make materials evaporate in vacuum or inert gas and then make atoms or molecules form nanoparticles, which is also suitable for the synthesis of cobalt oxide nanoparticles. During the synthesis of metal nanoparticles, ILs can act as stabilizers or structure-directing agents to control the shape, size and crystal structure. Schuster et al. reported the formation of cobalt oxide nanoparticles mediated by ozone in a low-temperature anhydrous ionic liquid environment, which led to the formation of cobalt oxide nanoparticles with a narrow size distribution and an average size of 4 nm [32].

Yu et al. produced cobalt oxide nanoparticles by pulsed laser fragmentation (PLFL) in a flowing water system. They used coffee grounds as a sustainable hard template to prepare spinel cobalt oxide through a simple method. Then, a mild reduction process was used to obtain cobalt oxide while maintaining a mesoporous structure composed of about 8 nm particles [33]. It was an effective method to construct cobalt oxide nanoparticles in a flowing water system, which was conducive to the sustainable production of active cobalt catalysts.

### 2.4. Green Biosynthesis

In recent years, the biosynthesis of nanoparticles has become a popular and mature technology due to their advantages in energy saving. Researchers have prepared cobalt oxide nanoparticles used in different fields through biosynthesis for different purposes [34,35]. As a safe, eco-friendly and simple method, biosynthesis mainly uses biological resources to synthesize cobalt oxide nanoparticles with various activities.

Plant extracts are very important components for the synthesis of cobalt oxide nanoparticles. Due to the abundant and safe nature of plants, the preparation of cobalt oxide nanoparticles by plant-mediated biosynthesis is very promising for their further medical uses. Hasan et al. separately mixed the pink cobalt chloride solution with 12 different solvent-based plant extract fractions and successfully synthesized cobalt oxide nanoparticles with different plant extracts as stabilizers [36]. Similarly, Kiani et al. synthesized cobalt oxide nanoparticles through the reaction of typical Spanish sage and cobalt nitrate [37]. Moreover, Raeisi et al. synthesized cobalt oxide nanoparticles by plant extracts from fresh leaves of rosemary twigs [38]. Phytochemicals in the extract of Rhamnus virgata leaf also have the functions to act as reducing and stabilizing agents, which can thus be applied to produce cobalt oxide nanoparticles [18].

Similar to plant synthesis pathways, bacteria can also be used as raw materials for the synthesis of nanoparticles. Different kinds of bacteria were used to synthesize cobalt oxide nanoparticles. A Gram-positive bacterium Bacillus subtilis was used to synthesize cobalt oxide nanoparticles by adding CoCl_2_·6H_2_O to the bacillus solution and adding NaBH_4_ to reduce it. After room-temperature incubation, centrifugation, washing and drying, the cobalt oxide nanoparticles were finally prepared [39]. In addition, Allen et al. also found that Listeria could also be used to synthesize cobalt oxide nanomaterials, which could improve the properties of mineral and protein components [40]. Compared with chemical methods, this method is more environmentally friendly, but its process is lengthy and can easily cause pollution.

As a kind of microorganisms, fungi can be used to synthesize cobalt oxide nanoparticles just like bacteria. VijayaNandan et al. mixed the fungal biomass with the cobalt acetylacetonate solution and stirred it under certain conditions to extract the nanoparticles in the supernatant [41]. The sterile silk filtrate of Aspergillus brasiliensis ATCC 16404 can also be used to synthesize cobalt oxide nanoparticles with a nanometer size range of 20 to 27 nm [42]. Because raw materials are easy to obtain and separate, this method has unique advantages for preparing cobalt oxide nanoparticles.

From the past to the present, people have been trying to use biomolecules to synthesize chiral inorganic nanoparticles. Kim et al. used L-and D-Tyr-Tyr-Cys ligands to synthesize chiral cobalt oxide nanoparticles with a factor of 0.01 in the ultraviolet-visible region. In short, the cobalt chloride, sodium citrate and Tyr-Tyr-Cys aqueous solution were mixed and stirred at room temperature for 30 min. Then NABH_4_ was injected, followed by aging under stirring for 2 h at room temperature to obtain cobalt oxide nanoparticles [43].

**Figure 1:**
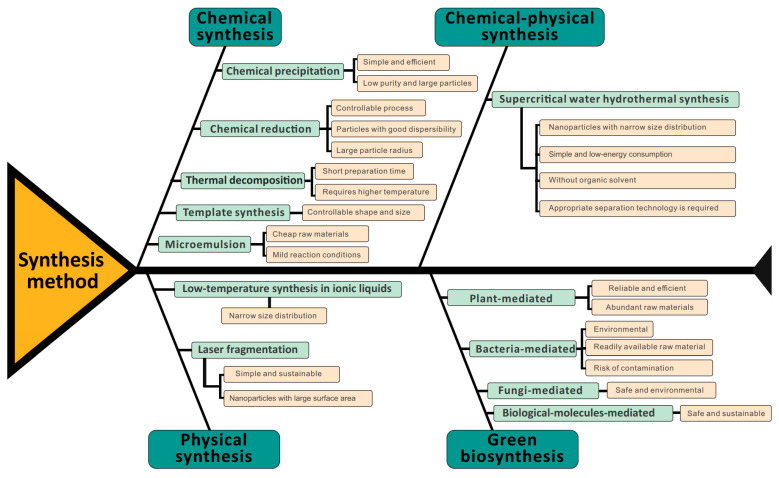
Typical synthesis method for cobalt oxide nanoparticles.

The synthesis of functional cobalt oxide nanoparticles has been highlighted as the most important issue for the future biomedical application of cobalt oxide nanoparticles. The basic methods for cobalt oxide nanoparticle preparation have been summarized in this work as shown in Table 1 and Figure 1. The basic principles, advantages and limitations of each method, as well as the size and structure of their product, are also indicated in Table 1 and Figure 1, which may be helpful for researchers to choose the most suitable strategy for cobalt oxide nanoparticle preparation. Current anticancer treatment strategies based on nanomedicines always require specific targeting effects and high selectivity against cancer cells, which therefore urges us to explore more methods for cobalt oxide nanoparticle preparation. We believe that the increasing development of the cobalt oxide nanoparticle preparation methods would finally benefit their future uses for anticancer treatment.

## 3. Biocompatibility of Cobalt Oxide Nanoparticles

The biocompatibility of nanomaterials needs to be carefully considered in their biomedical application. The toxicity of cobalt-containing nanoparticles always depends on the cobalt phase. Al Samri et al. found that, compared with zero-valent cobalt, the cobalt oxide phase has relatively low toxicity, better biocompatibility and more advantages for in vivo applications, which suggests the increased potential of cobalt oxide nanoparticles for biomedical uses [44]. In anti-tumor treatments, the biocompatibility of nanoparticles directly affects the anti-tumor effects, which therefore reminds us that it is particularly important to further increase the biocompatibility of cobalt oxide nanoparticles. Gold nanoparticles and silica nanoparticles are considered to have good biocompatibility [45,46], but they can also be quickly eliminated in the body through the reticuloendothelial system (RES). Some researchers have improved the biocompatibility of gold nanoparticles by modifying them with marine carbohydrates [47,48]. Similarly, the biocompatibility of cobalt oxide nanoparticles may also be improved by surface modification of some active molecules with high biocompatibility. The biocompatibility of cobalt oxide nanoparticles can also be improved by forming composite materials with other materials, for example, using silica as the protective shell of cobalt oxide nanoparticles, which significantly increases their biocompatibility [44]. In addition, similar to what Khan et al. reported [49], the biocompatibility and bioavailability of cobalt oxide nanoparticles can also be improved through the combined use of cobalt oxide nanoparticles and other nano preparations. Additionally, cobalt oxide nanoparticles obtained by different preparation methods have different biocompatibility. Therefore, it is very important to choose a suitable preparation method to obtain cobalt oxide nanoparticles with good biocompatibility, which would finally benefit the anticancer application of cobalt oxide nanoparticles.

## 4. Anticancer Applications of Cobalt Oxide Nanoparticles

Before the introduction of cobalt oxide nanoparticles for anticancer treatment, it is necessary to know the initial anticancer activities of cobalt and cobalt complexes. Cobalt is an essential trace element in all animals that shows lower toxicity than other non-essential metals, which suggests that cobalt may have great potential for further anticancer studies [50]. It has been proved that cobalt complexes can bind with DNA to cause spiral elongation, and a cytotoxicity analysis of the MCF-7 breast cancer cell line indicated that some compounds can act as powerful anticancer candidates [51]. Here we briefly summarize the potential anticancer applications and deficiencies of some cobalt complexes, including cobalt–Schiff-base complexes, cobalt–carbonyl complexes and cobalamin (Table 2). Although these cobalt complexes have shown attractive anticancer activities, there are still some critical issues that need to be further improved, which include the unknown pharmaceutics, stability, tumor hypoxia selectivity and systemic toxicity in vivo. More importantly, all these cobalt complexes did not show any advantages for targeted anticancer treatment, which needs to be further improved. However, the introduction of cobalt oxide nanoparticles highlights their potential applications for targeted anticancer treatment based on the established targeting ligands or antibody modification of cobalt oxide nanoparticles. Additionally, these cobalt complexes with different coordination molecules show wide anticancer activities, which also suggest some implications for the use of cobalt oxide nanoparticles for anticancer treatment as indicated in Table 2. It is hoped that the anticancer advantages of some cobalt complexes may also be extended for the improvement of cobalt oxide nanoparticles.

Under the pressure of increased mortality and obvious side effects in patients with the current anticancer strategies, it is urgent to explore novel cancer treatment strategies with high efficacy and low side effects. Since the 1980s, nanomaterials have been studied as functional drug carriers by enhancing the targeting effects and anti-tumor effects of drugs in vivo. After more than 30 years of exploration, it has been discovered that some nanomaterials based on nanomedicines have the advantages of high biocompatibility, high targeting and low toxicity for cancer treatment [52]. While many metal oxide nanoparticles have been proved to have anticancer activity [53], cobalt oxide nanomaterials have also been recently reported for their anticancer activities, which indicated their potential for anticancer treatment.

### 4.1. Selective Cancer Cell Inhibition by Cobalt Oxide Nanoparticles

The use of phosphine derivatives combined with metal atoms on the surface of metal nanoparticles can overcome toxicity problems by forming a hydrophilic, biocompatible and biodegradable coating on nanoparticles with stable binding [55]. Chattopadhyay S et al. combined methyl iminodiacetic acid phosphate with cobalt oxide nanoparticles (PMIDA-CoO) and investigated the effects of this substance on human leukemia cell lines (Jurkat, K562 and KG1A cells) [56]. In vitro results show that PMIDA-CoO·NPs could cause DNA damage in Jurkat, KG-1A and K562 cells as the PLD secreted by cancer cells would cause PMIDA-CoO NPs to release more cobalt ions for cancer cell inhibition [56]. Interestingly, PMIDA-CoO NPs can also reduce the release of cobalt ions in normal cells but with no inhibition effects on normal cells, which shows their selectivity against cancer cells [56]. However, it is worth mentioning that PMIDA-CoO can also induce high levels of TNF-α; therefore, the safe dosage of PMIDA-CoO needed to be further investigated in mice models as the body can be damaged by long-term and high levels of TNF-α exposure [57].

In addition to the anticancer treatment of cobalt oxide nanoparticles encapsulated with N-(phosphonomethyl) iminodiacetic acid in vitro, S. Chattopadhyay et al. used chitosan as a carrier to connect Co_3_O_4_ nanoparticles (CS-CoO) to deliver cobalt oxide nanoparticles to human leukemia cells (Jurkat cells). CS-CoO nanoparticles can induce cancer cell apoptosis by activating caspase-3, -8 and -9 and inducing the secretion of TNF-α [58]. The toxicity of CS-CoO nanoparticles to Jukat cells was dose-dependent with 50% maximum inhibitory concentration on Jurkat cells calculated to be 138.16 mg/mL. It is worth mentioning that Co_3_O_4_ NPs are toxic to lymphocytes when the concentration is higher than 100 μg/mL. Although chitosan-coated nanoparticles are insoluble under normal physiological pH, the concentration should be less than 100 μg/mL when used in biomedicine for safety reasons [58]. This is consistent with the experimental results of Arsalan N et al. who also proved that Co_3_O_4_ NPs can reduce the survival rate of K562 cells by damaging cell membrane, activating caspase-9, -8 and -3, increasing Bax/Bcl ratio and promoting ROS production, cell cycle arrest and apoptosis and do not affect the survival rate of normal lymphocytes [59].

**Figure 2:**
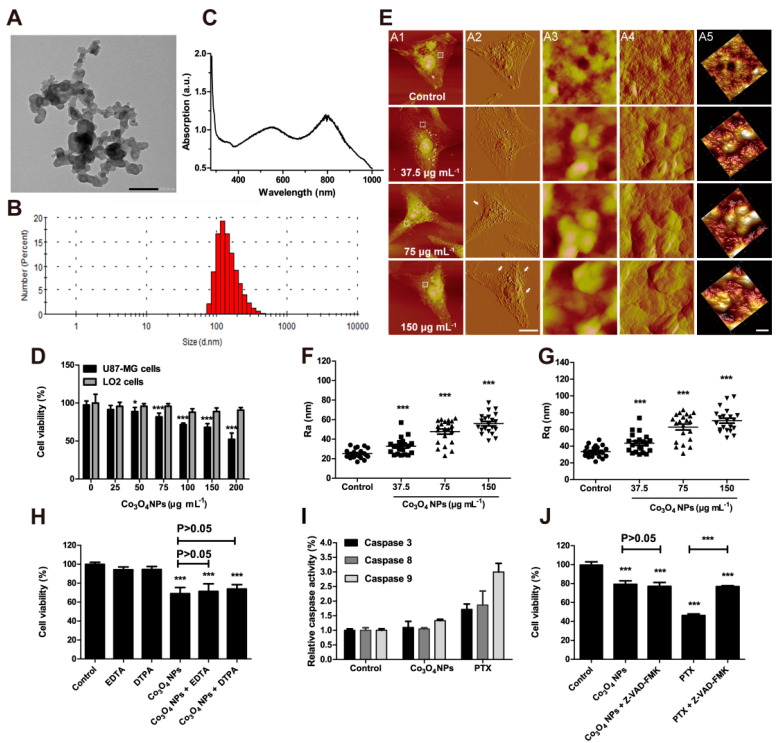
Co_3_O_4_ NPs induce non-apoptotic cytotoxicity against U-87 MG cells in a cobalt-ion-independent manner. (**A**) TEM image of Co_3_O_4_ NPs. Scale bar = 200 μm. (**B**) Size distribution of Co_3_O_4_ NPs. (**C**) UV-Vis-NIR absorption spectrum of Co_3_O_4_ NPs. (**D**) Viability of U-87 MG cells and LO2 cells after 48 h Co_3_O_4_ NP treatment. N = 6/group, * *p* < 0.05, *** *p* < 0.001. (**E**) AFM imaging of U-87 MG cells after Co_3_O_4_ NPs treatment, scale bar is whole cell imaging (two left panels) is 20 μm and the scale bar for ultrastructure imaging (three right panels) is 500 nm. (**F**) Average roughness (Ra) and (**G**) root-mean-squared roughness (Rq) analyzed from ultrastructure images of U-87 MG cells. N = 20/group, *** *p* < 0.001. (**H**) Viability of U-87 MG cells following treatment with Co_3_O_4_ NPs and/or the ion collators, EDTA or DTPA. N = 8/group, *** *p* < 0.001. (**I**) Caspase activity in U-87 MG cells following treatment with Co_3_O_4_ NPs or the apoptosis inducer, PTX. N = 3/group. (**J**) Viability of U-87 MG cells following treatment with Co_3_O_4_ NPs and/or the caspase inhibitor, Z-VAD-FMK. N = 4/group, *** *p* < 0.001. Values represent means ± S.D. Reproduced with permission from [20]. Copyright Elsevier, 2021.

Human beings have been fighting stomach cancer for a long time, and it is very good news that the incidence of gastric cancer in the world is falling year by year. However, the concern is that once the patient is diagnosed with gastric cancer, the survival rate is very low as it has the third-highest death rate worldwide [60]. Compared with individual drug therapy, the drug-loaded nano system has been proved to show better inhibition effects in stomach cancer cells [61]. Jarestan. M. et al. obtained Co_3_O_4_@Glu/TSC NPs by contraction and investigated their cytotoxicity on gastric cancer cells [21]. The obtained nanocomplex is coated with glutamic acid to show better moisture properties. In addition, the combination of thiosemicarbazide and nanoparticles can significantly improve the chemical activity of metal ions to strengthen the inhibition of cancer cells, thus showing synergistic cancer inhibition. More importantly, gastric cancer cells and normal cells were treated with the same concentrations of Co_3_O_4_@Glu/TSC NPs, which showed selective inhibition in gastric cancer cells [21]. Our recent work also demonstrated the use of Co_3_O_4_ NPs for anticancer treatment, which showed inhibition effects on U-87 MG cancer cells without any effects on LO2 normal cells (Figure 2) [20]. We also explored the potential cytotoxicity of Co_3_O_4_ NPs against U-87 MG cancer cells, which demonstrated that Co_3_O_4_ NPs inhibited U-87 MG cancer cell proliferation was not cobalt-ion- or apoptosis-dependent (Figure 2) [20]. These results indicate that a cobalt oxide nanoparticle combined complex could selectively inhibit the growth of cancer cells, which provides a novel potential for the development of more effective anticancer therapeutics with fewer side effects. However, their exact anticancer mechanisms are still needed to be further investigated.

### 4.2. Cobalt Nanoparticles as Chemosensitizer and Protective Agents for Anticancer Treatment

Neuroblastoma (NBL) is a malignant tumor caused by the failure of embryonic neural crest cells to differentiate. As NBL patients are very young, always infants, and more than three-quarters of NBL cases are diagnosed before the age of five [46], the commonly used chemotherapy drug for adult cancer treatment is very inappropriate for these cases. At present, cisplatin is a relatively common clinical treatment against cancers, including NBL [62]. However, researchers have found that cisplatin can make mitochondria more susceptible to reactive oxygen species (ROS) and lead to oxidative damage in normal cells. Long-term oxidative damage can lead to organ failure, in which the kidney is mostly injured [63]. Thus, it is very important to improve the treatment efficiency and reduce the side effects of cisplatin for its long-term use in cancer treatment.

In recent years, researchers have proved that cobalt nanoparticles can effectively reverse the side effects of cisplatin. Ertugrul MS et al. found that CoS nanoparticles–cisplatin bio-conjugates could significantly improve the anticancer effects of cisplatin, which shows the potential to minimize the dosage of cisplatin for anticancer treatment and therefore reduce cisplatin-associated side effects [64]. These results strongly suggest that cobalt nanoparticles had strong anticancer effects by acting as chemosensitizer and protective agents, which also indicated the potential of cobalt oxide nanomaterials to act as chemosensitizer and protective agents for anticancer treatment.

### 4.3. Cobalt Oxide Nanoparticles Re-Influence the Cancer Microenvironment

Macrophages act as the first-line immune cells to infiltrate pre-invasive tumorous lesions and persist during their further development into invasive cancer. In the tumor environment, macrophages gradually develop as key regulatory factors by their polarization. Based on the polarization pattern of M1 and M2 macrophages, inhibition of M2 and stimulation of M1 are considered to be a feasible strategy to restore the anticancer function of tumor-associated macrophages (TAMs) [65].

Nanoparticles can interact with the immune cells and therefore affect the immune system [66]. Chattopadhyay S et al. reported that PMIDA-CoO was combined with the antigen (CL) of oral cancer cell (KB) to form the Cl-PMIDA-CoO complex, which could stimulate macrophages for the activation of anticancer immune responses. It was found that pulsed macrophages had killing effects on KB cells with few inhibition effects on lymphocytes [25]. It could re-influence the cancer microenvironment by affecting TAM polarization, thereby significantly inhibiting the growth of cancer cells. Based on TAM activation, this PMIDA-CoO system provides a new immunological strategy for cobalt oxide nanoparticles to fight cancer [25].

At present, the principle of immunotherapy for cancer can be simply understood as an attempt to enhance host immune responses for tumor inhibition. Therefore, antigen-coupled Co_3_O_4_ nanoparticles can be effectively used in cancer immunotherapy and become an alternative strategy for cancer treatment. However, the disadvantage of the above immunotherapy is that only a few tumor-related specific antigens can be coupled with nanoparticles. S. Chattopadhyay et al. evaluated the anticancer immune response in vivo by combining the complete cleavage antigen of Dalton’s lymphoma with PMIDA-CoO nanoparticles, and the results show that PMIDA-CoO as a carrier of tumor vaccine could effectively activate the anticancer factors of the immune system, showing great potential as a carrier system. In addition, the vaccine-mediated immune response was enhanced by the use of adjuvants. Traditional vaccines use inorganic adjuvants with varying degrees of side effects, and the experimental results show that it is expected to become a new adjuvant for the preparation of the anticancer vaccine [67]. The results of cytokines show that Ag-PMIDA-CoO nanoparticles resist tumor cells by stimulating macrophages to induce TNF-α and activate other apoptotic factors. Injection of Ag-PMIDA-CoO nanoparticles in vivo can increase the immunostimulatory factor IFN-γ, decrease the immunosuppressive factor IL-10, significantly increase Th1 cytokines and CD4+ cells and induce immune response dominated by Th1. It is worth mentioning that it can significantly inhibit the development of the tumor by blocking the p38 signal pathway. Therefore, p38MAPK inhibitors can be used to improve the immune effect of macrophage-based anticancer vaccines. For Ag-PMIDA-CoO-induced IgG response, the transformation of IgG to IgG2a also showed the ability to induce Th1 response. However, the IgG antibody produced by immunization with Ag-PMIDA-COO can cause specific ADCC in the anti-Ag-PMIDA-COO immune serum (the main role of the host’s defense against tumor). In in vivo experiments in tumor mice, Ag-PMIDA-CoO effectively reduced the tumor volume and prolonged the life span of mice, which may be related to the activation of CD8+ T cells by macrophages [67].

The tumor cell lysates used in the above experiments contain a variety of antigens and can be presented to T cells because a certain type of cell lysate Ag may not induce effective T-cell responses in another type of cancer infection. The advantage of tumor lysates is that different types of cancer cells express various antigens. Therefore, this kind of multi-epitope immunity has more advantages than single-antigen or isolated-antigen, which is also one of the main development directions of the anti-tumor vaccine at present.

Another recent development direction of the anti-tumor vaccine is to load RNA, peptides or proteins on metal oxide nanoparticles as nano-vaccines. For example, ES et al. combined Co_3_O_4_ NPs with anticancer peptide (ACP) and anticancer RNA (ACR) for potential immunotherapy of melanoma, lung cancer and other cancers [68]. These attempts using Co_3_O_4_ NPs as an antigen delivery system indicate the attractive prospect of cobalt oxide nanoparticles for the development of novel anticancer vaccines.

### 4.4. Anticancer Application of Cobalt Oxide Nanoparticles as Autophagy Inhibitors, Chemosensitizers and Photosensitizers for Synergetic Anticancer Treatment

Autophagy, a self-degradation process to maintain cellular homeostasis through degradation-misfolded proteins or damaged organelles by the lysosomal system, has been found to be associated with various pathological conditions [69]. The vital role of autophagy played in tumor formation and progression has been widely accepted. Emerging studies have revealed that autophagy is a beneficial progress for cancer cell metabolism and can also serve as a pro-survival mechanism in cancer cells against multiple therapeutic stresses by accelerating the self-repairing of damaged cells and weakening the killing effects of anticancer therapeutics [70]. Thus, synergistic modulation of autophagy that augments cancer cell sensitivity to diverse therapies is proposed as a novel strategy for more effective cancer therapy [71]. In addition, the direct modulation of autophagy to disturb the autophagy processes either by autophagy inducers or autophagy inhibitors could serve as an anticancer strategy [72]. Interestingly, large amounts of nanomaterials, as autophagy inducers or autophagy inhibitors, have been found to modulate autophagy and have also been exploited as therapeutic agents for cancer treatment [73].

**Figure 3:**
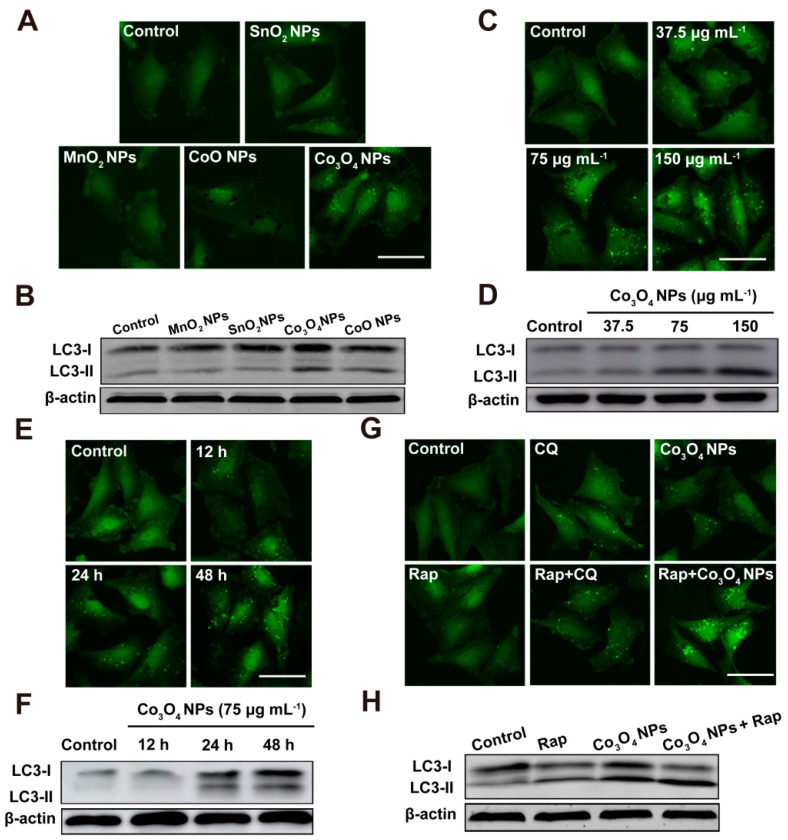
Co_3_O_4_ NPs treatment increases autophagosome formation and autophagy-related protein expression in U-87 MG cells. (**A**) Fluorescent imaging and (**B**) representative Western blots of U-87 MG/GFP-LC3 cells incubated with different kinds of NPs for 24 h. (**C**) Fluorescent imaging and (**D**) representative Western blots of U-87 MG/GFP-LC3 cells incubated with different concentrations of Co_3_O_4_ NPs for 24 h. (**E**) Fluorescent imaging and (**F**) representative Western blots of U-87 MG/GFP-LC3 cells with Co_3_O_4_ NPs for different durations. (**G**) Fluorescent imaging and (**H**) representative Western blots of U-87 MG/GFP-LC3 cells incubated with Co_3_O_4_ NPs and/or Rap for 24 h. Green puncta represent autophagosomes. Scale bar = 50 μm. Reproduced with permission from [20]. Copyright Elsevier, 2021.

Our recent work indicated that Co_3_O_4_ NPs inhibited U-87 MG cancer cell proliferation was not cobalt-ion- or apoptosis-dependent [20]. Therefore, we further investigated the role of autophagy in Co_3_O_4_ NPs inhibited U-87 MG cancer cell proliferation, which proved that cobalt oxide nanoparticles could selectively induce glioma cell death by enhancing autophagosome formation and autolysosome accumulation. To further confirm the exact inducer or inhibitor role of Co_3_O_4_ NPs in autophagy, we used autophagy inducer rapamycin (Rap) or autophagy inhibitor chloroquine (CQ) as control for mechanism studies, which indicated that Co_3_O_4_ NPs might act as a kind of autophagy inhibitor (Figure 3). We also further evaluated a range of autophagy-related proteins, including mTOR, p-mTOR, ATG5, ATG12 and Beclin-1 in glioma cells. However, the expression of mTOR, p-mTOR, ATG5, ATG12 and Beclin-1 in glioma cells was not increased after Co_3_O_4_ NPs treatment, which confirmed that Co_3_O_4_ NPs did not induce autophagy. After the comparison with rapamycin, an autophagy inhibitor, Co_3_O_4_ NPs were finally confirmed to be a kind of novel autophagy inhibitors. Further results show that the cytotoxic effect of Co_3_O_4_ NPs on U-87MG glioma cells affected the function of lysosomes by inhibiting the proteolytic activity of CTSB, resulting in impaired autophagy degradation. By destroying actin to prevent autophagy flow and reduce intracellular ATP level, the cell fuel cycle was reduced by Co_3_O_4_ NPs, resulting in the decrease of lysosomal proteolysis activity. Furthermore, Co_3_O_4_ NPs can inhibit the degradation of autosomes, induce the aggregation of autophagosomes, break the intracellular homeostasis and block the flux of autophagy to induce cell toxicity. The expression of p62, an autophagy substrate responsible for transporting ubiquitin, was also significantly increased by Co_3_O_4_ NPs treatment.

As one of the most important protein degradation pathways for cell metabolism and homeostasis, the ubiquitin–proteasome system (UPS) has been found to have the potential to be designed as a target for nano biotic anticancer therapies. UPS not only promotes the degradation of organelles in eukaryotic cells but also plays critical roles in the degradation of key proteins in tumor cells. Carfilzomib (CFZ) is a widely used clinical protease inhibitor that blocks cancer cell protein degradation by inhibiting UPS signaling. Autophagy is another critical protein degradation pathway to maintain cellular homeostasis, and more and more evidence has indicated that cancer cells could initiate protective autophagy to avoid the killing effects of anticancer drugs. Based on this situation, cancer cells would activate the autophagy upon CFZ treatment to clear the accumulated UPS substrates that are harmful to cancer cells, thus weakening the anticancer effects of CFZ. As Co_3_O_4_ NPs could serve as a novel autophagy inhibitor, the combination of Co_3_O_4_ NPs and CFZ could simultaneously impair the UPS and autophagy signaling, which would therefore block both protein degradation pathways in cancer cells. Specifically speaking, in this anticancer strategy, CFZ could block UPS signaling to induce accumulated UPS substrates, and simultaneously, Co_3_O_4_ NPs could block the protective autophagy flux of cancer cells to prompt the accumulation of autophagy substrate, which results in large amounts of undergraduate protein accumulation to disrupt cell homeostasis and lead to synergistic cancer cell death. This work clearly demonstrates the appealing applications of Co_3_O_4_ NPs as an autophagy inhibitor and chemosensitizer for synergetic anticancer treatment.

**Figure 4:**
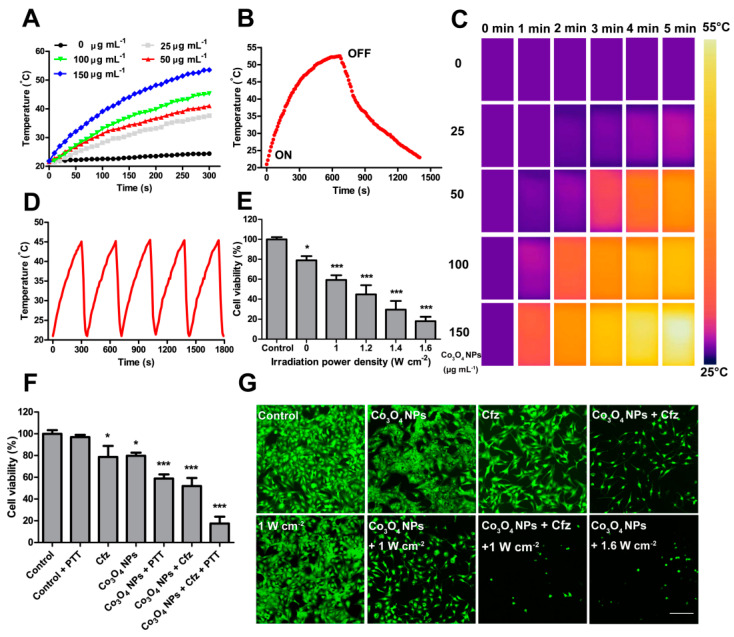
Co_3_O_4_ NPs and photothermal therapy synergize to enhance the anticancer efficacy of Cfz in U-87 MG cells. (**A**) Temperature of the Co_3_O_4_ NPs in aqueous solution over a period of 5 min exposure to laser irradiation (808 nm, 1 W·cm^−2^) and (**B**) after cessation of laser irradiation. (**C**) Infrared thermal images of the Co_3_O_4_ NPs in aqueous solution over a period of 5 min exposure to laser irradiation (808 nm, 1 W cm^−2^). (**D**) Temperature of the Co_3_O_4_ NPs in aqueous solution over 5 cycles of laser irradiation (808 nm, 1 W cm^−2^). (**E**) Viability of U-87 MG cells after 75 μg mL^−1^ Co_3_O_4_ NPs photothermal therapy at various laser power intensities (0−1.6 W cm^−2^) for 5 min, N = 4/group, * *p* < 0.05, *** *p* < 0.001. (**F**) Viability of U-87 MG cells after treatment with Co_3_O_4_ NPs and/or photothermal therapy and/or Cfz. N = 9/group, * *p* < 0.05, *** *p* < 0.001. (**G**) Representative calcein-AM staining of U-87 MG cells after treatment with Co_3_O_4_ NPs and/or photothermal therapy and/or Cfz. Scale bar = 200 μm. Values represent means ± S.D. Reproduced with permission from [20]. Copyright Elsevier, 2021.

**Figure 5:**
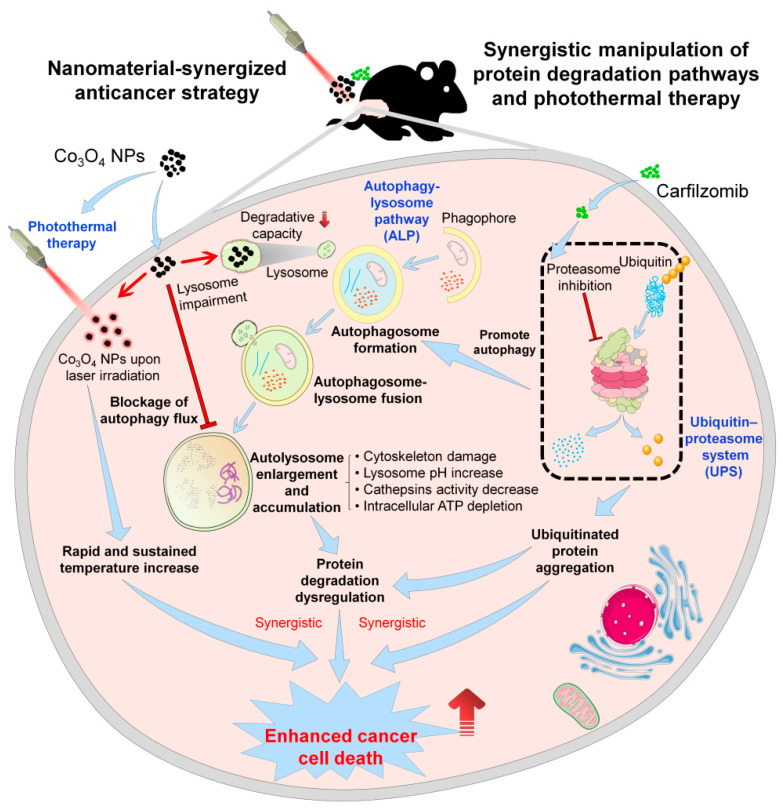
Co_3_O_4_ NPs synergized strategy manipulating autophagy, proteasome and photothermal therapy for enhanced anticancer therapeutics. Reproduced with permission from [20]. Copyright Elsevier, 2021.

In addition, photothermal therapy (PTT) has gained increasing attention due to the thermal ablation of cancer cells [74]. We previously demonstrated that Co_3_O_4_ NPs showed a very high photothermal conversion rate at 808 nm (40.48%) [20] compared to that of other PTT-stimulated metals (e.g., copper selenide nanocrystals 22% [75]; gold nanoparticles 37% [76]). The good photothermal conversion rate and photothermal stability of Co_3_O_4_ NPs further extended their use as a photosensitizer for cancer cell killing [20]. Furthermore, we combined PPT with the above combination (Co_3_O_4_+CFZ), which showed the highest inhibition on the viability of glioma cells in vitro, indicating the synergistic anticancer effects of the Co_3_O_4_+CFZ+PPT strategy (Figure 4) [20]. Moreover, consistent with our in vitro observation, the strongest inhibition of tumor growth was observed in Co_3_O_4_NPs+CFZ+PTT treated mice with no significant toxicity. This Co_3_O_4_ NPs synergized anticancer strategy combining the autophagy–lysosome pathway, ubiquitin–proteasome system and photothermal therapy with relatively low risk may potentially serve as a more effective anti-tumor therapeutics (Figure 5).

### 4.5. Cobalt Oxide Nanoparticle Based Drug Delivery Systems for Anticancer Treatment

One of the most accepted applications of nanomaterials is their potential to act as a vehicle for drug delivery [77]. In order to use Co_3_O_4_ NPs as drug carriers, it is necessary to verify whether they affect the structure of proteins when they are coupled with proteins, as the drug delivery system would directly interact with the proteins after administration. Human serum albumin (HSA) is the most abundant protein in plasma and is often used to carry a variety of drugs to meet various treatment requirements. After Arsalan et al. used different concentrations of Co_3_O_4_ NPs to interact with HSA molecules, the fluorescence results show that high concentrations of Co_3_O_4_ NPs could change the quaternary structure of HSA but did not affect the natural structure of HAS [59].

Chattopadhyay S et al. linked N-Phosphonomethyliminodiacetic acid (PMIDA) coated cobalt oxide nanoparticles (PMIDA-CoO) with folic acid (FA) for cancer-cell-targeted delivery of anticancer drugs, such as doxorubicin and methotrexate. Flow cytometry analysis confirmed that anticancer drugs loaded onto the complex showed high cytotoxicity and could induce apoptosis of cancer cells [28].

In recent years, researchers have been working on the development of a highly specific drug delivery system, nanoscale drug delivery system (NDDS) [78,79], which can reach the designated tumor microenvironment (TME) to improve the efficacy of drugs and achieve tumor imaging. At present, inorganic materials are commonly used in the NDDS, but the shortcomings of inorganic materials are also obvious, such as that they are non-biodegradable, not easy to remove from the body and so on. Therefore, at present, it is a research hotspot to explore drug carriers with higher specificity and removability in vivo.

Q. Ren et al. proposed to use the simple redox reaction between cobalt oxide and monodisperse hollow manganese to form adjustable hollow MCO NPs and encapsulate tumor therapeutic compounds (such as doxorubicin). Because the TME has a high concentration of glutathione (GSH), it can promote the release of DOX and achieve good tumor inhibition [80]. The experiment proved that the loading rate of MCO NPs to DOX is as high as 210%, and MCO can be completely degraded in GSH solution with high concentration. Incidentally, MRI has proved that MCO NPs can be used as a GSH responsive contrast agent [80]. Therefore, MCO NPs shows a good prospect in acting as a drug carrier and MRI contrast agent.

### 4.6. Other Applications of Cobalt Oxide Nanoparticles in Cancer Field

In addition, in terms of tumor markers, Dai H et al. designed an enzyme-free integrated biological probe composed of a heme-modified magnetic NiCo_2_O_4_ superstructure (ATS-MNS-HB) to achieve high sensitivity and high accuracy in the detection of tumor markers [81]. It shows the application prospect of cobalt oxide nanoparticles for accurate diagnosis strategy development of tumor markers. Zhang et al. used the chelation between the six histidine residues (His-Tag) at the C-terminal of the epidermal growth factor receptor ((EGFR) sdAb (EGFR sdAbs) and the divalent cobalt ions coated on the surface of Co_3_O_4_ NPs to form a new nano-probe. Finally, the obtained nano-probes were applied to the immunohistochemical (IHC) detection of EGFR expression in (NSCLC) tissues of non-small cell lung cancer with good stability and high sensitivity and specificity [82]. This work clearly demonstrates the development prospect of cobalt oxide nanoparticles to prepare nano-probes for the detection of cancer-associated markers.

Squamous cell carcinoma antigen (SCCA) is favored by many researchers as a tumor marker in different kinds of cancers [83], but it is difficult to detect SCCA in human serum. Li et al. have tried to use nanomaterials to amplify the signal, thus establishing a new type of non-enzyme sandwich electrochemical immunoassay method by constructing Co_3_O_4_@CeO_2_-Au@Pt complex to detect SCCA [84]. Firstly, the sandwich electrochemical immunosensor has a high sensitivity for the detection of SCCA. Secondly, Co_3_O_4_@CeO_2_ nanoparticles have high catalytic performance and electron transport ability, while sea-urchin gold–platinum nanoparticles (Au@Pt nanoparticles) have high stability and strong biological binding force, which can be combined to construct the nanocomposite Co_3_O_4_@CeO_2_-Au@Pt. By the cooperation of Co_3_O_4_, CeO_2_-Au, Pt and D-Au nanoparticles, the detection limit of SCCA was determined as 33 fg/mL with very good reproducibility [84]. The above test results suggest that this method might serve as a new idea for the clinical detection of tumor markers.

Finally, the interaction between nanoparticles and proteins may hinder or amplify the activity of enzymes. DeLong et al. used Co_3_O_4_ NPs to interact with two typical enzyme models: luciferase (Luc) and galactosidase (β-Gal) [85]. The results show that Co_3_O_4_ NPs significantly activated β-Gal, which indicated that biochemical activity was unique between enzymes and cobalt oxide nanoparticles. In addition, it is proved that the complex formed by nanoparticles with Luc, β-Gal or other labeled enzymes can be used to label cancer cells with photoluminescence (PL [85]). Thus, there might be some promising applications of cobalt oxide nanoparticles to regulate the activity of enzymes, which may also be useful for anticancer treatments.

## 5. Conclusions and Future Perspectives

**Figure 6:**
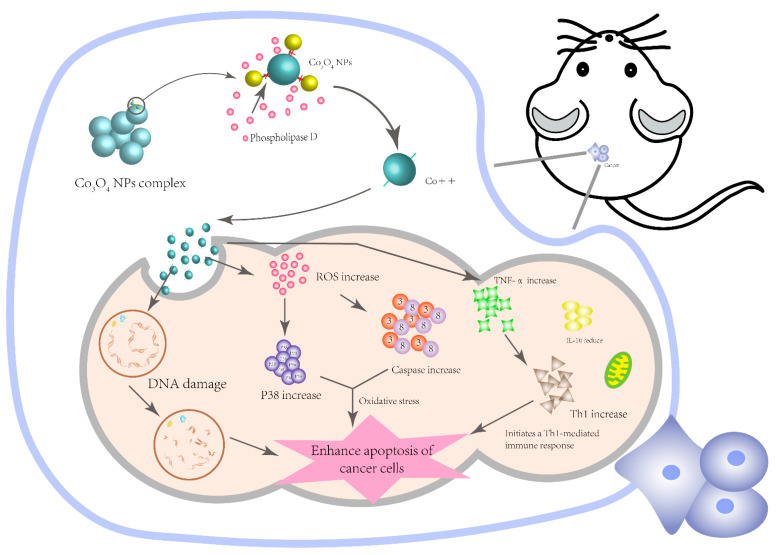
Potential mechanisms of cobalt oxide and its complex on cancer cell apoptosis.

Up to now, cobalt oxide nanoparticles have shown great potential for anti-tumor application with increasing attention. Based on the above information, tumor cell membranes contain more phospholipids than normal cells. The presence of large amounts of phospholipids may affect the attachment of CS-CoO and PMIDA-CoO nanoparticles to the membrane of cancer cells, and the presence of acids in the phospholipids may help the nanocomplex release free cobalt ions into cells, thereby allowing cell apoptosis by activating different biochemical processes [58]. The positively charged cobalt ions are more internalized into cancer cells, causing different degrees of DNA damage and activating oxidative stress and inflammation to promote apoptosis of cancer cells. Oxidative stress promotes the production of ROS, which in turn promotes the production of caspase-3 and -8 and activates the p38 signal pathway for apoptosis induction [67]. We also summarize the potential mechanisms of cobalt oxide and its complex on cancer cell apoptosis as shown in Figure 6.

Cobalt oxide nanoparticles can be directly used as anticancer drugs to induce cancer cell damage and can also be used as chemical sensitizers and protective agents during anticancer therapy, which may be very helpful to enhance the anticancer effects and reduce the side effects of chemotherapeutics. Additionally, cobalt oxide nanoparticles also have a good performance in improving the effects of immunotherapy as anticancer vaccines. Interestingly, with very good photothermal conversion abilities, cobalt oxide nanoparticles could also act as photothermal agents for photothermal therapy of cancer. Moreover, similar to most nanoparticles, cobalt oxide nanoparticles could also act as drug carriers for cancer-cell-directed drug delivery, which may significantly improve the cancer-targeting effects of anticancer drugs. Moreover, the ability of cobalt oxide nanoparticles to construct nano-probes or biosensors can be applied for cancer-associated molecule detection, while the regulation effects of cobalt oxide nanoparticles on enzyme activity also indicate their potential uses as enzyme regulators during cancer treatment.

At present, most anticancer studies focusing on cobalt oxide nanoparticles use a low concentration of nanoparticles to treat cancer cells. The lowest concentration that has killing effects on cancer cells but no killing effects on normal cells is always used as the effective concentration for anticancer application. However, some scholars have found that although normal cells at these concentrations do not die, there is a certain extent of growth inhibition. Thus, there is an urgent need to further study the toxicity mechanisms of cobalt oxide nanoparticles both in cancer cells and normal cells at certain doses. Wang et al. treated normal cells with a sublethal concentration of Co_3_O_4_ NPs and found that Co_3_O_4_ NPs did not cause obvious necrosis of normal cells [86]. However, Co_3_O_4_ NPs could interact with mitochondria, affect the expression of mitochondria-related genes, result in impaired mitochondrial function and decreased ATP production, which seriously inhibit the growth of normal cells. The mechanism of Co_3_O_4_ NPs to kill cancer cells was proved to be generally associated with plasma membrane damage or ROS accumulation, but this study did not find significant changes in ROS level and plasma membrane in normal cells, which needs to be further explored to explain these mechanisms [86]. At present, there are few studies on the biological effects of cobalt oxide nanoparticles at sublethal doses, and thus it is necessary to determine the concentration of cobalt oxide nanoparticles more accurately before they are expanded for clinical anticancer treatments, which would be helpful to minimize the side effects on normal cells.

The understanding of cobalt ion toxicity is very important to the use of cobalt-related materials for biomedical purposes. More mechanism studies are needed to explore the cytotoxicity of cobalt ions in different cancer cells and normal cells before the consideration of using cobalt oxide nanoparticles for clinic trials. With regard to the anticancer study of chitosan-coated Co_3_O_4_ NPs, in fact, more cobalt ions were released by chitosan-coated Co_3_O_4_ in acidic conditions than that in neutral pH conditions, and this effect was positively correlated with the cytotoxicity of the nanoparticles [87]. Therefore, it is also important and necessary to further study the cobalt ion release behaviors of cobalt oxide nanoparticles when they act as drug carriers under different pH values and physiological conditions, especially in vivo.

The concept of using nano-carrier systems for cancer-targeted drug delivery is still restricted by the development of functional targeting ligands and drug-encapsulating materials. For the drug carrier roles of cobalt oxide nanoparticles, how to construct functional cobalt oxide nanoparticle delivery systems with outstanding cancer-cell-targeting effects would be the most important issue. However, how to encapsulate drugs into cobalt oxide nanoparticles with high loading rates also remains a critical issue for their further applications.

The surface properties of nanoparticles have been well known to play critical roles in their biomedical applications. In addition, it is worth noting that how the shape and size of cobalt oxide nanoparticles affect their anticancer activities should also be an important subject for the study of cobalt oxide nanoparticles. Raman et al. found that Co_3_O_4_-NP-B (bulk) has a stronger inhibitory effect on liver glutathione (GSH) activity than Co_3_O_4_-NP-S (spherical) [26]. This work shows that, while paying attention to the surface area of nanoparticles, we should also strengthen the exploration of the influence of shape and toxicity for cobalt oxide nanoparticles.

Up to now, although some reports about cytotoxicity of cobalt oxide nanoparticles against normal cells have been demonstrated, there are still no reports describing the biocompatibility of cobalt oxide nanoparticles, which is critical for their further clinical uses. Therefore, more attention should be paid to the biocompatibility of cobalt oxide nanoparticles, such as their hemolytic properties and sensitization responses.

As one of the trace elements, cobalt is essential for normal physiological activities of the body, especially for its role as a metal constituent of vitamin B12. However, we cannot deny that excessive exposure to cobalt would induce various adverse health effects, causing unavoidable damage to the body and affecting hearing, vision, nervous, cardiovascular and endocrine systems [88,89]. Therefore, the use of cobalt oxide nanoparticles for anticancer treatment still needs to be further carefully investigated, especially its potential cytotoxicity in vitro and system toxicity in vivo. Additionally, the metabolism of cobalt oxide nanoparticles in vivo should be paid more attention in future research of cobalt oxide nanoparticles for biomedical uses, as it directly affects the adverse effects of nanoparticles in the body. We also believe that, despite the potentiality of the therapeutic approach of cobalt oxide nanoparticles, the use of cobalt for treating cancer should be carefully employed due to side effects in healthy tissues.

In our opinion, the best future development of cobalt oxide nanoparticles for anticancer therapy should be focused on the following: (i) the anticancer mechanism exploration of cobalt oxide nanoparticles; (ii) the drug delivery roles of cobalt oxide nanoparticles against cancer; (iii) the roles of cobalt oxide nanoparticles in rebuilding tumor microenvironment; (iiii) the ability and mechanisms of cobalt oxide nanoparticles to sensibilize current chemotherapy; (iiiii) the development of cobalt oxide nanoparticles for photothermal therapy; (iiiiii) the evaluation of potential adverse effects induced by cobalt oxide nanoparticles; and (iiiiiii) the systemic metabolism of cobalt oxide nanoparticles in vivo. With the increased attention paid to these fields, we believe that we can build a strong network to estimate the potentials of cobalt oxide nanoparticle based anticancer strategies.

Overall, although lots of work is still needed to further extend cobalt oxide nanoparticles for anticancer treatment, the promising abilities of cobalt oxide nanoparticles indeed present strong potential to fight cancer by acting in different roles. With the increasing attention paid to cobalt oxide nanoparticles, we believe that there would be more and more strategies coming out for anticancer treatments based on cobalt oxide nanoparticles. The continued development of functional cobalt oxide nanoparticles would finally benefit the current therapies for cancer, as well as other biomedical fields.

## Figures and Tables

**Table 1:** Typical synthesis method for cobalt oxide nanoparticles.

Synthesis Method	Principle	Advantages and Limitations	Typical Reference	Size (nm)	Structure or Shape
Chemical precipitation	The cobalt precursor and the precipitant solution are mixed and stirred first, then centrifuged, dried to collect the precipitates and then heated to obtain cobalt oxide nanoparticles.	This method is simple and easy. The prepared nanoparticles have high antibacterial and anticancer activity; however, the prepared nanoparticles have low purity and large particle radius.	[7]	27	Spinel structure
			[19]	20−25	Non-spherical, irregular-shaped
			[10]		Spherical, cuboidal or hexagonal
			[24]	<100	Quasi-spherical
			[26]	40−60	Spherical
Chemical reduction	The reducing agent is chemically reduced with the metal salt solution.	The particles are basically spherical with good dispersibility. The process can be controlled, but the particle radius is large.	[27]	60 ± 6	Spherical
Thermal decomposition	Compound is decomposed at high temperature to prepare cobalt oxide nanopowder.	This method can quickly produce cobalt oxide nanoparticles but requires a higher temperature.	[23,25,28]	<100	Spherical
Template synthesis	Using the voids in the matrix as a template for synthesis.	This method obtained nanoparticles with uniform size and periodic configuration in space.	[29]	~6	Hollow
Microemulsion method	The immiscible solvents form emulsions under the action of surfactants and form nuclei in the “microbubbles”, coalesce, agglomerate and heat-treat to obtain nanoparticles.	The nanoparticles have small average particle size and high stability.	[30]	1−5	Spherical
Supercritical water hydrothermal synthesis	In the critical state, the gas and liquid phases of the removed liquid no longer coexist during the solvent removal process, thereby eliminating surface tension and capillary as a force to prevent the gel structure from collapsing and agglomerating and obtaining ultrafine oxides.	The prepared cobalt oxide nanoparticles have high surface area and have adjuvant activity under certain conditions.	[31]	6.9−32.5	NA
Low-temperature synthesis in ionic liquids	Ionic liquids are used as stabilizers or structure-directing agents to synthesize nanoparticles with specific shapes, sizes and structures in a low-temperature environment.	This method avoids high-temperature calcination and hydroxide formation, resulting in a narrow size distribution of nanoparticles.	[32]	4	Monocrystalline
Laser fragmentation	Cobalt oxide nanoparticles are produced by pulsed laser fragmentation of liquid (PLFL) in a flowing water system.	It is simple and sustainable, and the produced nanoparticles have high surface area and strong catalytic activity.	[33]	<5	Spinel structure
Plant-mediated synthesis	Using plants as raw materials to prepare nanoparticles through co-precipitation, fractionation, typical reactions, etc.	The method is reliable, efficient and reduces the physical and chemical burden. The prepared nanoparticles have good catalytic activity, anti-microorganism, antifungal or anticancer activity.	[18]	<100	
			[34]		Rhombus-shaped
			[36]	<100	Bead-shaped, crystal-shaped or cube-shaped
			[37]	41 ± 3.0	Nearly spherical
			[38]	~10	Cubic-phase structure
Bacteria-mediated synthesis	Using bacteria or bacterial components as templates to synthesize nanoparticles at different temperatures.	The method is environmental. Raw materials are easy to obtain but may introduce contamination.	[39]	~5	Rod-shaped
			[40]	5	Crystal-shaped
Fungi-mediated synthesis	Fungi are used as reducing media to synthesize nanoparticles in the presence of precursor solutions.	This method is safe and environmentally friendly. Fungi are easy to obtain and cultivate.	[41]	10−30	Predominantly spherical
			[42]	20−27	Quasi-spherical
Biological-molecules-mediated synthesis	Nanoparticles were synthesized using biomolecules as medium.	A safe, environment-friendly and sustainable preparation method. The prepared nanoparticles have good biocompatibility and catalytic activity.	[43]	<50	Spinel

**Table 2:** Summary of the application of cobalt oxide complexes.

Complex Form	Cobalt Complexes	Application	Insufficient	Implications for Nano Cobalt Oxide	Reference
Cobalt–Schiff-base complex	Cobalt (II) complex containing 2,6-bis (2,6-diethylthio-aminomethyl) pyridine	Anti-colorectal adenocarcinoma (HCT-15) and cervical adenocarcinoma (HELA) cells	1. The biological activities of cobalt–Schiff-base complexes vary, requiring systematic structure–activity relationship studies to determine the true pharmaceutical potential of these compounds. 2. Although this compound has a good performance in inducing DNA damage and apoptosis of cancer cells, the use of concentration still needs to be studied.	/	[50]
	Cobalt (III) complex containing three Schiff-base ligands derived from the reaction of salicylaldehyde and ethylenediamine	Anti-human breast cancer cells (moderate activity)		/	
Cobalt–carbonyl clusters	Co-ass [Co_2_(CO)] complex with acetylene-containing aspirin derivatives	When combined with imatinib, it showed inhibition of proliferation of acute and chronic myelogenous leukemia cells	1. Weak ability to inhibit cancer cells. 2. The anticancer potential and in vitro COX inhibition of these compounds cannot be evaluated because no cell-based studies have been reported.	/	[50]
	Cobalt (II) complexes bind to non-steroidal anti-inflammatory drugs (NSAIDs)	They showed strong affinity for biphasic DNA and HSA		/	
Preparation of hypoxia selective prodrugs	Cobalt(III)-mustard agents	Hypoxic cancer cells	In vivo studies showed metabolic instability and high systemic toxicity.	1. Given that many tumors are acidic and hypoxic, this strategy may be very useful in the development of new tumor-specific delivery systems. 2. The complexation of curcumin ligand to CO(III)-TPA enables better provision of cobalt protein, uniform delivery of curcumin ligand throughout the tumor model and free curcumin that accumulates in the outer edge.	[50]
	Cobalt (III) complexes as bioactive ligands		A longer administration time is needed to test the true efficacy of the complex.		
	CO (III)-1,4,7,10-Tetraazadecane (cyclonin) complex		Stability and hypoxia selectivity need to be improved.		
	CO (III) -cyclam-azaCBI		In view of the test results, the identity of the reductase or non-enzymatic reductant responsible for reducing these complexes is still unknown.		
	Cobalt (III) complex for imaging hypoxic areas		Cobalt (III) complexes are thought to produce unstable cobalt (II) complexes and the release of one or more biologically active ligands. However, the exact mechanism that activates this process is still elusive.		
Cobalt-containing cobalamin	Cobalamin-Chlormagnesium Biological Heterocyclic Protein	Nano-toxicity of breast cancer and melanoma cancer cells	IC50 value is 10 times higher than free serum curve.	Nitric oxide (NO) transfers to cancer cells in the form of nitrocobalamin. Extensive cytotoxic studies have shown that nitrosocobalamin preferentially kills cancer cells. It has good application prospects in hard-to-reach areas in the body.	[50]
	Cobalamin	Used to transport small gas molecules of high significance	/		
Diphenylhydrazine cobalt (II) complex	Co(II)complex [Co(bpy)(az)_2_](PF_6_)2 and [Co(az)_4_](PF_6_)	Apoptosis of SKHEP-1 cells was induced	The pharmacokinetics of the complex against cancer need to be tested to detect changes in the proteins associated with apoptosis of cancer cells.	/	[54]
